# A Vaccine Targeting Ovine Herpesvirus 2 Glycoprotein B Protects against Sheep-Associated Malignant Catarrhal Fever

**DOI:** 10.3390/vaccines10122156

**Published:** 2022-12-15

**Authors:** Cristina W. Cunha, Katherine N. Baker, Donal O’Toole, Emily Cole, Smriti Shringi, Benjamin G. Dewals, Alain Vanderplasschen, Hong Li

**Affiliations:** 1 Animal Disease Research Unit, Agricultural Research Service, USDA, Pullman, WA 99164, USA; 2 Department of Veterinary Microbiology and Pathology, Washington State University, Pullman, WA 99164, USA; 3 Department of Veterinary Sciences, University of Wyoming, Laramie, WY 82070, USA; 4 Fundamental and Applied Research in Animals and Health (FARAH), Immunology-Vaccinology, Faculty of Veterinary Medicine, University of Liège, 4000 Liège, Belgium

**Keywords:** malignant catarrhal fever, ovine herpesvirus-2, vaccine, viral-vector, DNA immunization

## Abstract

Malignant catarrhal fever (MCF) is a complex and often fatal disease of ungulates. Effective vaccines are needed to avoid MCF outbreaks and mitigate losses. This study aimed to evaluate a sheep-associated MCF (SA-MCF) vaccine candidate targeting ovine herpesvirus 2 (OvHV-2) glycoprotein B (gB). Rabbits were used as a laboratory animal model to test the safety, immunogenicity, and protective efficacy of a chimeric virus consisting of a recombinant, non-pathogenic strain of alcelaphine herpesvirus-1 encoding OvHV-2 ORF8 to express gB (AlHV-1^∆ORF73^/OvHV-2-ORF8). Viral-vectored immunizations were performed by using the AlHV-1^∆ORF73^/OvHV-2-ORF8 chimera alone or as a DNA prime (OvHV-2-ORF8)-virus boost regimen. The viral vector was inoculated by intravenous or intramuscular routes and the DNA was delivered by intradermal shots using a gene gun. The vaccine candidates were deemed safe as no clinical signs were observed following any of the immunizations. Anti-OvHV-2 gB antibodies with neutralizing activity were induced by all immunogens. At three weeks post-final immunization, all animals were challenged intranasally with a lethal dose of OvHV-2. MCF protection rates ranging from 66.7% to 71.4% were observed in vaccinated rabbits, while all mock-vaccinated animals developed the disease. The significant protective efficacy obtained with the vaccine platforms tested in this study encourages further trials in relevant livestock species, such as cattle and bison.

## 1. Introduction

Malignant catarrhal fever (MCF) is an often-fatal lymphoproliferative syndrome of ungulates caused by a group of gamma-herpesviruses in the genus *Macavirus* referred to as MCF viruses (MCFV) [1,2,3,4]. Among the MCF viruses, ovine herpesvirus-2 (OvHV-2) and alcelaphine herpesvirus-1 (AlHV-1) are most frequently associated with disease in livestock. These viruses are closely related, and cause diseases that are clinically and pathologically similar, but have different host tropisms and induce no cross-reacting neutralizing antibodies [5,6,7]. AlHV-1 is prevalent in Africa, where it is carried by wildebeest and causes wildebeest-associated MCF (WA-MCF) in cattle [8]. OvHV-2 is carried by sheep worldwide and is responsible for cases of sheep-associated MCF (SA-MCF) in several species, including cattle, bison, deer, and pigs [9]. SA-MCF is particularly important to the bison industry due to the high susceptibility of bison to the disease, resulting in devastating outbreaks when bison and sheep are maintained in proximity [10,11]. Preventing transmission of OvHV-2 is key to protecting clinically susceptible populations. Currently, the only way to control the disease is by separating carrier and susceptible hosts. However, since the effective separation of animals can be impractical and challenging in some agricultural settings, vaccination of the susceptible animals presents an alternative and desirable solution to mitigate the economic impact of animal losses due to MCF.

Development of a vaccine to protect susceptible animals from MCF has been a priority for several research groups in the field [12,13,14,15,16]. An attenuated strain of AlHV-1, obtained after successive passages in cell culture [17], has been tested as a vaccine to WA-MCF with promising results. Vaccine trials testing the attenuated AlHV-1 vaccine in cattle resulted in protection rates of up to 90% following AlHV-1 experimental challenge or exposure to wildebeest [18,19,20,21,22,23]. For SA-MCF, novel approaches need to be considered, since there is no culture system to propagate OvHV-2. Our group is working on the development of chimeric viruses capable of delivering key OvHV-2 proteins as immunogens. We demonstrated that treatment of OvHV-2 with antibodies specific to OvHV-2 envelope glycoprotein (g) B and/or the heterodimer gH/gL renders the virus unable to infect rabbits, the laboratory model used to study OvHV-2 infection and SA-MCF development [24]. In follow-up studies, a vaccine based on a recombinant bovine herpesvirus-4 (BoHV-4) expressing OvHV-2 gB was developed and tested in rabbits. The vaccine trial in rabbits resulted in 43% of protection from disease following the challenge with OvHV-2 [15]. Although the vaccine platform and regimens need improvement to increase efficacy, the trial supports the use of OvHV-2 gB as a potential target for protective immune responses. 

In this study, we tested the OvHV-2 gB target in a new SA-MCF vaccine platform based on an attenuated AlHV-1 that lacks the gene encoding the latency-associated nuclear antigen (ORF73). This virus, AlHV-1^∆ORF73^, is infectious but unable to induce MCF and establish persistent infection in vivo [25]. Using this virus as a backbone, we have previously produced an AlHV-1/OvHV-2 chimera by replacing AlHV-1 ORF8 with the OvHV-2 ORF8 [12]. Here, a rabbit model was used to evaluate the AlHV-1^∆ORF73^/OvHV2-ORF8 chimeric virus delivered either by prime and booster immunizations or as a heterologous prime-boost approach, where OvHV-2 ORF8 DNA immunizations were performed prior to the viral-vectored vaccine. Results of these trials indicated that the vaccine candidate is immunogenic and can induce protection following the OvHV-2 challenge. 

## 2. Materials and Methods

### 2.1. Animals

Thirty-eight New Zealand rabbits were used in the study. The animals were obtained from Western Oregon Rabbit Co., Portland, OR, and maintained at Washington State University according to approved Federal and State regulations and protocols for animal welfare and use. The minimal number of animals to allow meaningful conclusions were considered in the study. Upon arrival, the rabbits were acclimated for one week before beginning the experiments. Rabbits were randomly separated into groups as described in Table 1 as vaccinated [DNA+VV(IV), VV(IV), and VV(IM)] or mock-immunized. Rabbits were immunized at 11 weeks of age. Two 10-month-old animals (Mock-31, and -32) were used as controls for the second challenge to match the age of vaccinated animals in Experiment 1.

### 2.2. Vaccine Formulations

The chimeric virus used as a vaccine vector, AlHV-1^∆ORF73^/OvHV-2-ORF8, consists of the AlHV-1^∆ORF73^ viral genome as a backbone with its ORF8 gene replaced by the OvHV-2 homolog [12,25]. The chimera, obtained using homologous recombination, is cloned as a bacterial artificial chromosome (BAC) [12]. BAC cassette-excised AlHV-1^∆ORF73^/OVHV-2-ORF8 was used in this study and viral stocks were obtained using standard methods, as previously described [12,15]. Briefly, the BAC cassette was excised after 2–3 virus passages in immortalized fetal mouflon sheep kidney cells (FMSK_htert.1_) expressing Cre recombinase (FMSK_htert.1_/Cre). The virus was then propagated in FMSK_htert.1_ cells until approximately 80% cytopathic effect (3 to 4 days post-infection). Viral particles were harvested by freezing and thawing cultures three times, clarified by centrifugation, and stored at −80 °C until use. Virus preparations were titrated by a limiting dilution assay and the titers were expressed as TCID50/mL.

For DNA immunizations, we used a mammalian expression vector that contains a codon-optimized sequence of OvHV-2 ORF8 previously prepared in our laboratory [24]. This plasmid is identified as pOvHV-2-ORF8 and expresses OvHV-2 gB fused to the V5 epitope at the C-terminus end. Endotoxin-free pOvHV-2-ORF8 DNA was obtained from transformed *E. coli* using an EndoFree Plasmid Mega Kit (Qiagen, Hilden, Germany).

### 2.3. Immunizations and Challenge

Table 1 summarizes the immunization platforms tested in the study. For genetic immunizations, pOvHV-2-ORF8 was delivered into the skin using a gene gun (Helios^®^ Gene Gun System, Bio-Rad, Hercules, CA, USA), as previously described [24]. Rabbits were briefly anesthetized with isoflurane to reduce mobility and animal stress and a total of 12 µg of pOvHV-2-ORF8 coated on gold particles was bombarded on shaved abdominal skin at 400 psi helium pressure. AlHV-1^∆ORF73^/OvHV-2-ORF8 was delivered at a dose of 10^4.5^ TCID50 per injection via intravenous [1 mL injection, groups DNA+VV(IV) and VV(IV) in experiment 1] or intramuscular [1 mL injection in the hind limb muscles, VV(IM) in experiment 2] routes. Culture supernatant from uninfected FMSK_htert.1_ cells, prepared using the same procedures as for the viral formulation, was used as the mock-immunogen in the control rabbits (Mock in experiments 1 and 2). Rabbits in all groups received a prime and two booster immunizations according to the regimen indicated in Table 1. For experiment 1, immunizations were performed on days 0, 14, and 28, while rabbits in experiment 2 were immunized on days 0, 21, and 42.

Three rabbits from each group in experiment 1 were euthanized at 46 days post-prime immunization for evaluation of immune responses prior to the challenge. All remaining rabbits were challenged with a lethal dose of OvHV-2 at three weeks post-last immunization. The challenge was delivered intranasally by nebulization of 2 mL of OvHV-2 stocks containing 10^6^ viral genome copies. Viral stocks used in the challenges were obtained from sheep nasal secretions that were prepared, quantified, and stored as previously described [26]. The animals in groups DNA+VV(IV) and VV(IV) that survived the challenge received a second inoculation of OvHV-2 (10^6^ viral genome copies by intranasal nebulization) at 181 days post-last immunization (160 days post-first challenge). 

### 2.4. Animal Monitoring

Rabbits were monitored for clinical signs, viremia, and antibody response at specific time points throughout the experiment. Blood samples collected in EDTA from the ear central artery were obtained prior to prime immunization and at least weekly following immunizations and challenge to monitor antibody responses and viremia. OvHV-2 infected animals that developed clinical signs compatible with MCF were euthanized within 48 h of onset of fever (sustained rectal temperature >40 °C) or immediately if any other sign, such as depression, anorexia, adipsia, diarrhea, or oculo-nasal discharge was observed. The remaining rabbits were euthanized at the end of the experiments at 272 and 127 days post-prime immunization in experiments 1 and 2, respectively. For euthanasia, animals were anesthetized with ketamine and xylazine and then injected with an overdose of pentobarbital. Necropsy was performed on all rabbits immediately after euthanasia. Bronchial alveolar lavage (BAL) fluid was obtained by washing the trachea and lungs with 5–7 mL of PBS and collecting as much fluid as possible. BAL fluids were clarified by centrifugation and stored at −20 °C until used. Samples from lung, liver, and mesenteric lymph nodes were also harvested with adjacent samples either snap-frozen in liquid nitrogen for virus quantification or stored in 10% formalin. 

#### 2.4.1. Pathology

Formalin-fixed tissues were embedded in paraffin and processed for histology in hematoxylin/eosin-stained slides. Histological examinations were performed by a pathologist. Tissues with no lesions were indicated as no visible lesion (NVL), and lesions were scored by severity as mild (+), moderate (++), or severe (+++) according to an MCF lesion score reference previously described [27].

#### 2.4.2. Viral DNA Analysis

Viral DNA in blood and tissues was detected and quantified with two PCR assays specific for AlHV-1 and OvHV-2 as described [28,29,30]. Total DNA was purified using QIAamp DNA Mini Kit (Qiagen) and quantified using a fluorometer (Qubit^TM^ and Qubit dsDNA BR Assay Kit, ThermoFisher Scientific, Waltham, MA, USA) as per the manufacturers’ recommendations. PCR mixes (20 μL) consisted of 1X TaqMan Fast Advanced Master Mix (ThermoFisher Scientific), specific primers and probes (concentration and sequences indicated in Table S1), and 50 ng of total DNA. Cycling conditions for both PCR assays were: 50.0 °C for 2 min, 95.0 °C for 2 min, followed by 40 cycles of 95.0 °C for 15 s, 60.0 °C for 30 s, with a plate read after each cycle. A CFX thermocycler and the CFX manager software (Bio-Rad) were used for cycling and data analysis, respectively. For both PCRs, results are expressed as viral genome copies per 50 ng of total DNA.

#### 2.4.3. Antibody Response Analysis

OvHV-2 gB antibody responses were assessed by indirect enzyme linked-immunosorbent assay (ELISA) using a lysate of pOvHV-2-ORF8 transfected mammalian cells as antigen [15,24]. Plasma was diluted at 1:400 and BAL fluid at 1:20. For detection, a peroxidase-conjugated anti-rabbit IgG (H+L) (Jackson ImmunoResearch, West Grove, PA, USA) at 1:4000 or an anti-rabbit IgA (Invitrogen) at 1:10,000 was used as a secondary antibody, followed by the TMP Microwell Peroxidase Substrate (ThermoFisher Scientific). For both IgG and IgA OvHV-2 gB ELISAs, results were reported as a ratio of the tested sample OD to the background OD (blank).

Neutralizing antibodies in plasma were measured by a neutralization assay using a recombinant bovine herpesvirus 4 (rBoHV-4) that expresses OvHV-2 gB instead of its own gB (rBoHV-4/OvHV-2-gB). Briefly, plasma and BAL fluid were treated at 56 °C for 10 min, and then mixed with 10^2^ TCID50 rBoHV-4/OvHV-2-gB to a final dilution of 1:8. Following a 1 h incubation at 37 °C, the plasma and virus mixture was combined with FMSK_htert.1_ cells (2.5 × 10^4^ cells/well), placed into four wells of a 96-well plate and cultured for eight days at 37 °C and 5% CO_2_. Uninfected cells, cells incubated with an untreated virus (virus only), and virus treated with standard plasma samples, positive and negative for OvHV-2 gB antibodies, were included in each plate as assay controls. Following the final incubation step, the cells were fixed (10% formaldehyde), stained (0.1% crystal violet), and the number of plaques counted. Virus-neutralizing activity based on OvHV-2 gB was calculated as a percentage of inhibition using the formula: % inhibition = 100 − (X × 100/*Max*), where X is the average number of plaques in the tested plasma and *Max* is the number of plaques in the virus only control. 

### 2.5. Statistical Analysis

Vaccine efficacy in each group was evaluated by calculating protection rates following the experimental OvHV-2 challenge. Differences in protection rates in vaccinated and controls were analyzed with Fisher’s Exact Test. One-Way ANOVA followed by Tukey’s multiple comparisons test was used to analyze antibody responses in the vaccinated and control groups and the Mann-Whitney test (False Discovery Test for multiple comparisons) to compare antibody responses in vaccinated animals that were protected or developed MCF. For all statistical analyses, *p* < 0.05 was considered significant. GraphPad Prism (Version 9.1.1 for Windows) was used for statistical analysis and graph preparation.

## 3. Results

**Figure 1:**
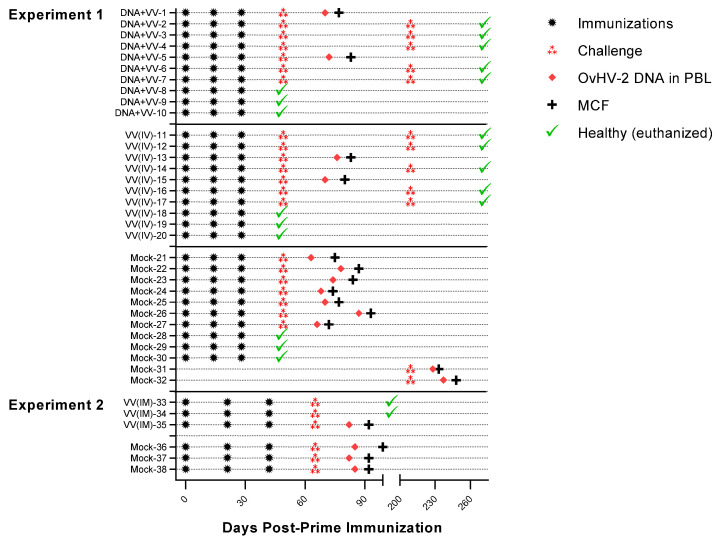
Schematic representation of treatments (immunizations and OvHV-2 challenge) and infection/disease outcomes.

Here we show the results of two experiments that evaluated the safety, efficacy, and immunogenicity of an SA-MCF vaccine candidate targeting OvHV-2 gB (Table 1). A schematic representation of immunization regimens, OvHV-2 challenge, and infection/disease outcomes is presented in Figure 1.

### 3.1. Safety and Protective Efficacy of OvHV-2-gB Vaccine Candidates

Following immunizations and prior to the challenge, no local or systemic adverse effects from vaccine delivery or clinical signs and mortality due to infection with the vaccine virus were observed in any of the rabbits, indicating that both the DNA formulation, delivered into the skin, and the chimeric virus, injected by either intramuscular or intravenous routes, were safe in this animal model.

The chimeric virus, AlHV-1^∆ORF73^/OvHV-2-ORF8, used as an immunogen in all vaccinated rabbits was not detected in the blood at any time post-vaccination or in tissues at the end of the experiment, as tested by AlHV-1 qPCR, confirming that the virus did not establish a latent infection in any of the rabbits. 

Vaccine efficacy was measured by comparing protection from MCF in vaccinated and unvaccinated (mock control) groups. As summarized in Table 2, five out of seven (71.4%) rabbits vaccinated either with DNA followed by the chimeric virus [DNA+VV(IV)] or with the virus only [VV(IV)] in experiment 1 were protected from disease after challenge with OvHV-2. In experiment 2, two out of three vaccinated animals [VV(IM)] (66.6%) were protected from disease following the challenge. Protection rates in unvaccinated rabbits [mock] in both experiments was 0% since all animals developed MCF after the challenge. This confirms that the dose of OvHV-2 used for the challenge was lethal to rabbits and that the animals that survived had protective immunity provided by the immunizations. Protection rates were significantly higher (*p* = 0.0210) in vaccinated groups than in the mock group in Experiment 1. For experiment 2, no statistically significant difference (*p* = 0.400) in protection rates in vaccinated and mock groups was observed, possibly due to the low number of animals in each group. All 10 rabbits in the DNA+VV(IV) and VV(IV) groups that received a second OvHV-2 challenge at 181 days post-last immunization remained healthy until the end of the experiment at 63 days post-challenge (DPC). The two rabbits that served as non-immunized controls in this challenge developed MCF (Figure 1, Mock-31 and -32). 

In the animals that developed MCF following the OvHV-2 challenge, the disease had a similar progression regardless of the vaccination status. In all OvHV-2 infected animals, the first day for detection of viral DNA in the blood ranged from 14 to 38 DPC, and no significant difference (*p* = 0.9508) was observed among immunized and mock groups. Similarly, no difference among groups (*p* = 0.9849) was observed for incubation time; in all groups, animals developed MCF from 23 to 44 DPC. There was also no difference in the levels of viral DNA in tissues (*p* = 0.5716, 0.4173, and 0.2510 for the lung, mesenteric lymph node, and liver, respectively). Individual values for the detection of viral DNA in blood, euthanasia due to MCF, viral genome copies, and lesion scores in tissues are presented in Table S2.

### 3.2. Immunogenicity Assessment of OvHV-2-gB Vaccine Platforms

**Figure 2:**
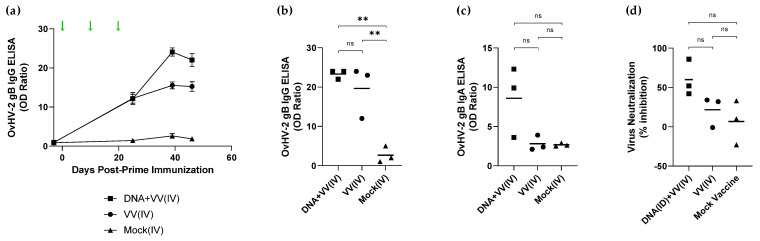
Anti-OvHV-2 gB antibody responses in three animals immunized with OvHV-2 DNA plus AlHV-1-vectored vaccine [DNA+VV(IV)], AlHV-1-vectored vaccine only [VV(IV)], or mock control [Mock(IV)] and euthanized prior to challenge in experiment 1. Anti-OvHV-2 gB antibodies of both isotypes were detected by ELISA assays. (**a**) IgG kinetics in plasma. Green arrows represent immunizations and error bars are SEM; (**b**) IgG in bronchial alveolar lavage (BAL) fluid; (**c**) IgA in BAL fluid, and (**d**) neutralizing antibody response in BAL fluid. BAL fluid was collected at 46 days post-prime immunization. Bars in panels (**b**–**d**) indicate mean; ns, no statistically significant difference between groups; **, *p* < 0.01.

Three rabbits from each group in experiment 1 were euthanized prior to challenge at 46 days post-prime immunization (DPI) for assessment of systemic and pulmonary antibody responses. Significantly higher levels of OvHV-2 gB-specific IgG antibodies were detected in both plasma (*p* < 0.001) and BAL fluid (*p* < 0.01) of the OvHV-2-gB immunized animals compared to the mock control (Figure 2a,b). Anti-OvHV-2 gB IgA was also measured in BAL fluid (Figure 2c). No OvHV-2-IgA response was observed in the VV(IV) group; the antibody levels in this group were similar to the mock group. Two rabbits in the DNA+VV(IV) group showed higher IgA levels as compared to the remaining animals in the same group or the other groups, but overall, no significant difference was observed among the groups (*p* = 0.0552). Neutralizing antibody activity based on OvHV-2 gB in BAL fluid was assessed by measuring the percentage of inhibition of a recombinant virus that uses OvHV-2 gB to infect cells. Percentage of inhibition ranging from 42 to 86% and from −1 to 34% were observed in the DNA+VV(IV) and VV(IV) groups respectively, while in the mock group percentage of inhibition varied from −23 to 33% (Figure 2d). No statistically significant difference was obtained among the groups, although animals in DNA+VV(IV) group tended to develop a more consistent neutralizing antibody response. 

**Figure 3:**
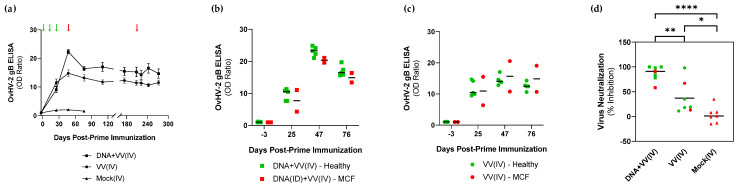
Anti-OvHV-2 gB antibody response in plasma of animals immunized with OvHV-2 DNA plus AlHV-1-vectored vaccine [DNA+VV(IV)], AlHV-1-vectored vaccine only [VV(IV)], or mock control [Mock(IV)] and challenged with OvHV-2 at 49 days post-prime immunization in Experiment 1. (**a**) Anti-OvHV-2 gB antibody responses pre- and post-immunizations and challenge measured by ELISA; error bars represent SEM and arrows represent immunizations (green) and challenges (red) times. (**b**,**c**) Individual anti-OvHV-2 gB antibody response in animals that remained healthy or developed SA-MCF following OvHV-2 challenge in the DNA+VV(IV) (**b**) and VV(IV) (**c**) groups; bars indicate the median. (**d**) Individual anti-OvHV-2 gB neutralizing antibody response measured by viral neutralization assay in each group at 46 days post-prime immunization; green symbols refer to animals that remained healthy after challenge and red symbols to animals that developed MCF; bars indicate mean; **** *p* ≤ 0.0001, ** *p* = 0.0022, and * *p* = 0.

The level of OvHV-2-gB-specific antibodies in animals immunized and challenged in experiment 1 is shown in Figure 3. Following immunization, antibody response levels in the OvHV-2-gB immunized groups increased up to 21.3 and 13.8 times in the DNA+VV(IV) and VV(IV), respectively, when compared to the mock, which showed no antibody response to OvHV-2 gB (*p* < 0.01 for both groups). Higher antibody levels were measured in the DNA+VV(IV) group compared to the VV(IV), especially at 47 DPI, but overall, there was no statistically significant difference between the two groups (*p* = 0.2471). Importantly, antibody levels remained high around 6 months post-immunization when animals received a second challenge and remained healthy (Figure 3a). When anti-OvHV-2 antibody titers were compared between animals that developed MCF or remained healthy after the challenge (Figure 3b,c), no difference was observed at any time point during the experiment (*p* > 0.05, all time points within each group). Neutralizing antibody responses were evaluated in plasma collected immediately prior to the challenge at 46 DPI (Figure 3d). Average percentages of inhibition of 87.0 ± 15.3 and 37.3 ± 33.0 were observed in the DNA+VV(IV) and VV(IV) groups, while in the mock group it was 3.4 ± 16.8 (*p* < 0.0001 and *p* = 0.0346 for the DNA+VV(IV) and VV(IV) groups, respectively, when compared to the mock). Although the average levels of neutralizing antibodies in the DNA+VV(IV) immunized group was higher than in the VV(IV) group (*p* = 0.0022), no difference was observed between immunized animals that developed MCF or remained healthy after challenge in any group (Figure 3d). 

**Figure 4:**
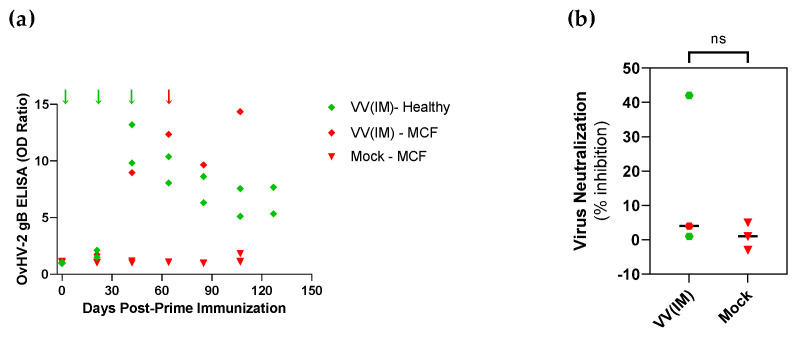
Anti-OvHV-2 antibody response in plasma of animals that developed MCF or were protected from disease following immunization with the AlHV-1-vectored vaccine delivered by intramuscular injection [VV(IM)] or a mock control and challenge with OvHV-2 in Experiment 2. (**a**) Individual OD ratio in plasma diluted at 1:400, as detected by ELISA. (**b**) Neutralizing antibody response at challenge time (64 days post-prime immunization), as measured by viral neutralization assay. Arrows represent immunizations (green) and challenge (red) times. ns, not statistically significant.

Antibody responses in rabbits that received the viral-vectored vaccine candidate delivered by intramuscular route (experiment 2) are shown in Figure 4. All three immunized rabbits developed anti-OvHV-2 gB IgG antibodies following immunizations (Figure 4a). Antibodies were detected in the blood only after the second booster immunization, with a peak in the levels of antibodies at 42 DPI, immediately before receiving the second booster (Figure 4a). At this time point, levels of antibodies in the immunized animals increased 9.6 times to pre-immunization levels, while in the mock group only a 0.3 increase was observed (*p* = 0.0220). Following the challenge, antibody titers declined and seemed to have reached a plateau around three months post-initial immunization. At 64 DPI, immediately prior to the challenge, neutralizing antibodies were observed only in one immunized rabbit, which showed a percentage of inhibition of 42% (Figure 4b).

## 4. Discussion

In this study, we used a well-established rabbit model for MCF studies to demonstrate that an AlHV-1-vectored OvHV-2 gB vaccine, delivered alone or as a heterologous DNA prime-viral boost immunization, is safe and can protect against OvHV-2-induced MCF. Both the AlHV-1^∆ORF73^/OvHV-2-ORF8 chimeric virus, inoculated by either intravenous or intramuscular injections, and the OvHV-2-ORF8 plasmid, delivered as a genetic immunization using a gene gun, resulted in no local or systemic adverse reactions. The safety of the immunogens for rabbits was expected based on previous studies showing that the chimeric virus is non-pathogenic due to the deletion of ORF73 [12,25] and that biolistic delivery of the plasmid pOvHV-2-ORF8 DNA does not cause any harm to rabbits [24]. Importantly, the vaccine candidates tested in different regimens or delivery routes resulted in protection against MCF upon challenge with a lethal dose of OvHV-2 in most of the vaccinated animals. 

The delivery routes used in Experiment 1, intravenous for the viral vector and intradermal for the DNA, were chosen because they have been previously used in the rabbit model with satisfactory results regarding induction of OvHV-2 gB-specific humoral immune responses [12,24]. Although delivering an immunogen by these routes may not be practical for vaccination, results obtained here will serve as guidance for further trials in target species, where vaccine delivery can be adapted to more suitable administration routes. Preliminary results obtained in this study using intramuscular injection (Experiment 2), indicate that this route of administration of the viral-vectored vaccine can also result in protection. 

Protection against SA-MCF has been previously demonstrated by using a recombinant BoHV-4 to express OvHV-2 gB [15]. Using the BoHV-4 vectored vaccine, 42.9% of the immunized rabbits were protected from MCF following OvHV-2 challenge. The BoHV-4-based chimeric virus contained both the BoHV-4 and the OvHV-2 genes encoding gB. This may have decreased the amount of OvHV-2 gB produced by the virus in vivo, since it was not essential for virus infection. In an attempt to improve vaccine efficacy, here we tested an alternative viral vector to deliver OvHV-2 gB. The new chimeric virus consists of a recombinant AlHV-1 that had ORF73 deleted and ORF8, encoding gB, replaced by the OvHV-2 ORF8. This chimeric virus used alone or following an OvHV-2 gB DNA prime immunization resulted in a better protection rate (66.6–71.4%) than the previously tested formulation (42.9%) following a challenge with the same dose of virus. The dose of OvHV-2 used for these studies is considered lethal for rabbits, as confirmed by the development of MCF in 100% of the mock-vaccinated animals in all vaccine trials. 

It has been previously demonstrated that neutralizing antibodies do not cross-react among MCF viruses [6], therefore humoral protective immune responses conferred by the SA-MCF vaccine candidates tested so far, vectored by BoHV-4 [15] and AlHV-1 (this study), were expected to be driven by their common antigen, OvHV-2 gB. However, the better levels of protection obtained when AlHV-1 was used as a vector in comparison to BoHV-4 suggest that potential shared T-cell epitopes between AlHV-1 and OvHV-2 may also have a role in protection. Although T cell-mediated immune responses are difficult to evaluate in the rabbit model due to the lack of reagents, a comprehensive analysis of immune response induced by AlHV-1^∆ORF73^/OvHV-2-ORF8 is planned for future experiments in cattle. 

The animals that remained healthy following the OvHV-2 challenge provide evidence that protective immune responses were successfully stimulated by the vaccine. While the type of immune responses needed to prevent MCF is not completely defined, studies using an attenuated AlHV-1 strain as a vaccine for AlHV-1-induced MCF in cattle show that protection is associated with the presence of neutralizing antibodies in the upper respiratory tract, while antibodies in the blood do not seem to play a role in protection [18,19,22]. Due to difficulties in consistently obtaining nasal secretion samples from live rabbits to evaluate immune responses in the respiratory tract, in Experiment 1 we euthanized three rabbits in each group prior to the challenge and used both plasma and BAL fluid to evaluate antibody responses. Interestingly, significant levels of OvHV-2 gB-specific IgG were detected both in plasma and BAL of immunized animals, while IgA measured in BAL was only detected in two animals in the DNA+VV(IV) group, which also showed consistent neutralizing antibody responses. These results indicated that mucosal antibody responses can be stimulated through vaccination, especially with DNA immunization prior to the viral-vectored immunization. By testing plasma pre- and post-challenge, we confirmed that systemic anti-OvHV-2 gB antibodies are not sufficient to protect against SA-MCF, as high levels of antibodies were observed in the blood of both protected and non-protected animals. Although correlates of protection could not be established in the trials performed in rabbits, the immune response detected indicates that the tested vaccine candidates were immunogenic and able to confer protection. Additional studies are needed to confirm and further explore the types and magnitude of immune responses induced by vaccination that are associated with protection. 

It is interesting to note that following OvHV-2 challenge, development of MCF in animals that were not protected progressed regardless of the immunization status of the animals, OvHV-2 gB vaccinated or unvaccinated. This corroborates the idea that once a certain viral load threshold is reached in peripheral blood cells, infection advances to disease in a similar fashion [31]. It has been shown in experimental settings that disease development following initial infection is dose-dependent and that different species have distinct susceptibilities to infection and disease [9]. For instance, an inoculum containing 10^4^ OvHV-2 genome copies is not enough to cause infection in rabbits, while an inoculum of 10^6^ is invariably fatal [32]. In this vaccine trial, all vaccinated animals that remained healthy after the challenge were not infected with OvHV-2, as demonstrated by the consistent absence of viral DNA in both the blood and tissues. This shows that protective immune responses induced by vaccination were able to reduce the viral load of the challenge inoculum to a dose that did not cause disease. Although sterile immunity may not be always necessary, depending on the species’ susceptibility to clinical disease, an efficacious SA-MCF vaccine should be able to maintain the viral load following OvHV-2 exposure at levels that are insufficient to cause infection or disease.

Lifelong immunity is a desirable factor in vaccines. This is particularly important for species that are not handled frequently, such as bison, one of the major targets for a SA-MCF vaccine. For the attenuated AlHV-1 vaccine, protection decreased to 50 and 37.5% when the challenge was performed at approximately 6 and 9 months after immunization, respectively [19]. To start addressing the issue related to the duration of immunity induced by the vaccines tested in this study, we submitted vaccinated animals that survived the first challenge to a second inoculation of the same lethal dose of OvHV-2 at 6 months after the last immunization. All five animals in each DNA+VV(IV) and VV(IV) groups survived the second challenge, suggesting that long-term immunity can be achieved, although additional studies evaluating extended time points are necessary. It is important to note that the first challenge in these animals resulted in no infection, therefore immunity was likely due to immunization and not from previous exposure to OvHV-2.

The recombinant AlHV-1 used as a backbone for our virus-vectored vaccine candidate can replicate in the host but is unable to persist as a latent virus due to the deletion of the gene encoding the virus’s latent-associated nuclear antigen [25]. Although there are advantages to live vaccines in which the viral vector can remain active following initial infection, it also offers concerns for safety, with a risk of virulence reversion [33,34]. The fact that the recombinant AlHV-1 does not persist may explain the low level of responses stimulated after prime and one booster immunization, requiring a third booster for higher antibody levels. An option to increase and/or modulate the immune responses induced by this vaccine is to add adjuvants with the recombinant virus. Attenuated AlHV-1 formulated with Emulsigen, an oil-in-water adjuvant, induces significantly higher levels of neutralizing antibodies than virus only [35]. Delivering AlHV-1^∆ORF73^/OvHV-2-ORF8 with an adjuvant may increase not only efficacy but also the duration of immunity. Studies to test this hypothesis are currently underway.

Rabbits were used as a laboratory animal model in this study as they were shown to be a suitable species to study SA-MCF [27]. While trials in this model are extremely valuable to screening potential vaccine candidates and evaluating protective immune responses, definitive vaccine trials to confirm safety and efficacy must be done in the animal species for which the vaccine is intended. The observation that the vaccine candidate tested in this study induced significant protection in rabbits paves the way for further research to investigate the effects of these vaccines in animals that are naturally susceptible to SA-MCF, including cattle and bison. Overall, vaccination resulting in protective immunity is one of the most effective methods of disease control and will ensure that animals susceptible to SA-MCF are protected from the disease even when in close contact with sheep carrying OvHV-2.

## 5. Conclusions

The results of this study demonstrated that the AlHV-1-vectored OvHV-2 gB vaccine delivered alone or in combination with OvHV-2 gB DNA immunization can protect animals from OvHV-2 infection and the development of SA-MCF. Importantly, we have demonstrated that injection of the viral-vectored vaccine by intramuscular route, a more practical route for vaccine administration, can also induce protection. Although we have not identified correlates of protection, the vaccine candidates were highly immunogenic as demonstrated by robust humoral responses, with high levels of specific antibodies against OvHV-2 gB, including neutralizing antibodies. These findings indicate that the AlHV-1-vectored OvHV-2 gB vaccine is a promising candidate warranting further investigation in relevant livestock.

## Figures and Tables

**Table 1:** Immunogens and vaccination platforms tested in this study.

	Group ^1^	Number of Rabbits	Vaccination	
			Immunogen ^2^	Regimen
Experiment 1				
	DNA+VV(IV)	10	pOvHV-2-ORF8 + AlHV-1^∆ORF73^/OvHV-2-ORF8	DNA prime and 1st booster, intradermal VV 2nd booster, intravenous
	VV(IV)	10	AlHV-1^∆ORF73^/OvHV-2-ORF8	VV prime and 2 boosters, intravenous
	Mock(IV)	10	Mock	3 inoculations, intravenous
Experiment 2				
	VV(IM)	3	AlHV-1^∆ORF73^/OVHV-2-ORF8	VV prime and 2 boosters, intramuscular
	Mock(IM)	3	Mock	3 inoculations, intramuscular

^1^ Vaccinated and mock. DNA (pOvHV-2-ORF8)VV, viral-vectored (AlHV-1^∆ORF73^/OvHV-2-ORF8); IV, intravenous; IM, intramuscular. ^2^ pOvHV-2-ORF8, 12 µg plasmid DNA/immunization; AlHV-1^∆ORF73^/OvHV-2-ORF8, virus at 10^4.5^ TCID50/immunization; Mock, uninfected FMSK_htert.1_ cell culture supernatant, 1 mL/immunization.

**Table 2:** Efficacy of OvHV-2-gB based vaccine candidates following OvHV-2 lethal challenge in a rabbit model.

	Experiment 1				Experiment 2	
	DNA+VV(IV)	VV(IV)	Mock		VV(IM)	Mock
Protected (Healthy)	5 (71.4%)	5 (71.4%)	0 (0%)		2 (66.7%)	0 (0%)
Unprotected (MCF)	2 (28.6%)	2 (28.6%)	7 (100%)		1 (33.3%)	3 (100%)
Total	7	7	7		3	3

DNA+VV(IV), OvHV-2 gB plasmid (prime and 1st booster), delivered intradermally using a gene gun, followed by the AlHV-1^∆ORF73^/OvHV-2-ORF8 vector delivered by intravenous (IV) injection. VV, AlHV-1^∆ORF73^/OvHV-2-ORF8 vector delivered by intravenous (IV) or intramuscular (IM) injections. Mock, immunization with uninfected cell culture supernatant.

## Data Availability

Data is contained within the article or Supplementary Materials. Additional data or raw data are available on request from the corresponding author.

## Supplementary Materials

The following supporting information can be downloaded at: https://www.mdpi.com/article/10.3390/vaccines10122156/s1, Table S1: Primers and probes used for OvHV-2 and AlHV-1-specific PCR assays; Table S2: Clinical parameters and outcome following immunization and OvHV-2 challenge. References [28,29,30] are cited in the Supplementary Materials.
