# Supplementary material for: A Vaccine Targeting Ovine Herpesvirus 2 Glycoprotein B Protects against Sheep-Associated Malignant Catarrhal Fever
Source: Vaccines (Basel). 2022 Dec 15;10(12):2156. doi: 10.3390/vaccines10122156 (PMC9786699; doi:10.3390/vaccines10122156)
Supplement: Supplementary file 1 [file vaccines-10-02156-s001.zip › vaccines-2076911-supplementary.pdf]

Table S1. Primers and probes used for OvHV-2 and AlHV-1-specific PCR assays.

Prime/Probe Name	DNA Sequence (5′-3′)	Final Reaction Concentration
AlHV1-F[1]	gggctaatttgtgcagtttgtga	50 nM
AlHV1-R[1]	aggtgtttctgaaaagaggggaa	300 nM
AlHV1-P[1]	6FAM/acaggctcctcgtcctcgtcgtgt/TAMRA	80 nM
OvHV2-F[2]	tggtaggagcaggctaccgt	240 nM
OvHV2-R[2]	atcatgctgacccccttgcag	600 nM
OvHV2-P[2]	56-FAM/tccacgccg/ZENtccacgccgtccgcactgtaaga/3IABkFQ	80 nM

[1] AlHV-1 specific qPCR (Traul, et al., 2005, https://doi.org/10.1016/j.jviromet.2005.05.021)

[2] OvHV-2 specific qPCR (Hussy, et al., 2001, doi: 10.1128/CDLI.8.1.123-128.2001; Traul, et al., 2007, https://doi.org/10.1177/104063870701900412)

Table S2. Clinical parameters and outcome following immunization and OvHV-2 challenge.

Immunogen[1]	Animal ID	Infection Outcome (DPC)		Clinical Status	OvHV-2 DNA levels[2] / Lesion Score[3]		
		Viremia[2]	Fever		Lung	Mes. LN	Liver
**Experiment 1**							
DNA+VV(IV)	1	21	26	SA-MCF	2x10^4 / +	3x10^2 / -	2x10^5 / ++
	2	ND	-	Healthy	ND / -	ND / -	ND / -
	3	ND	-	Healthy	ND / -	ND / -	ND / -
	4	ND	-	Healthy	ND / -	ND / -	ND / -
	5	23	33	SA-MCF	4x10^4 / +	2x10^3 / -	2x10^5 / +
	6	ND	-	Healthy	ND / -	ND / -	ND / -
	7	ND	-	Healthy	ND / -	ND / -	ND / -
VV(IV)	11	ND	-	Healthy	ND / -	ND / -	ND / -
	12	ND	-	Healthy	ND / -	ND / -	ND / -
	13	27	33	SA-MCF	4x10^4 / +	3x10^3 / -	4x10^4 / +
	14	ND	-	Healthy	ND / -	ND / -	ND / -
	15	21	30	SA-MCF	5x10^4 / +	1x10^3 / -	4x10^5 / +++
	16	ND	-	Healthy	ND / -	ND / -	ND / -
	17	ND	-	Healthy	ND / -	ND / -	ND / -
Mock(IV)	21	14	25	SA-MCF	9x10^3 / +	5x10^3 / -	1x10^5 / ++
	22	29	38	SA-MCF	4x10^3 / +	7x10^3 / -	5x10^3 / +
	23	25	34	SA-MCF	1x10^5 / +	6x10^3 / -	2x10^4 / ++
	24	19	24	SA-MCF	2x10^5 / +	4x10^3 / -	2x10^5 / ++
	25	21	28	SA-MCF	7x10^4 / +	3x10^2 / -	7x10^4 / ++
	26	38	43	SA-MCF	4x10^4 / +	3x10^3 / -	5x10^4 / +
	27	17	22	SA-MCF	3x10^4 / ++	2x10^4 / -	6x10^4 / +++
	28	19	24	SA-MCF	2x10^4 / +	2x10^3 / -	5x10^4 / ++
	29	28	39	SA-MCF	9x10^3 / +	1x10^3 / -	1x10^4 / +
**Experiment 2**							
VV(IM)	33	ND	-	Healthy	ND / -	ND / -	ND / -
	34	ND	-	Healthy	ND / -	ND / -	ND / -
	35	17	27	SA-MCF	1x10^4 / ++	2x10^5 / ++	8x10^4 / ++
Mock(IM)	36	26	34	SA-MCF	8x10^4 / +	2x10^4 / -	1x10^5 / +
	37	17	27	SA-MCF	1x10^5 / +	2x10^4 / -	2x10^5 / ++
	38	20	27	SA-MCF	2x10^5 / ++	1x10^4 / -	3x10^5 / +

[1] DNA+VV(IV), OvHV-2 gB plasmid (prime and 1st booster), delivered in the skin, followed by the AlHV-1$^{\Delta ORF73}$/OvHV-2-ORF8 chimeric virus delivered by intravenous (IV) injection. VV, AlHV-1$^{\Delta ORF73}$/OvHV-2-ORF8 chimeric virus delivered by intramuscular (IM) injection. Mock, immunization with uninfected cell culture supernatant.

[2] Detection of OvHV-2 DNA by PCR in blood. OvHV-2 qPCR expressed as OvHV-2 genome copies / 50 ng total (Hussy, et al., 2001, doi: 10.1128/CDLI.8.1.123-128.2001; Traul, et al., 2007, https://doi.org/10.1177/104063870701900412).

[3] Histopathological analysis, lesion scores: +, mild; ++, moderate; +++, severe. -, no visible lesion.

DPC, days post-challenge; ND, not detected; Mes. LN, mesenteric lymph node.

References:

Hussy, D.; Stauber, N.; Leutenegger, C.M.; Rieder, S.; Ackermann, M. Quantitative fluorogenic PCR assay for measuring ovine herpesvirus 2 replication in sheep. Clin. Diagn. Lab Immunol 2001, 8, 123–128.

Traul, D.L.; Taus, N.S.; Oaks, J.L.; O'Toole, D.; Rurangirwa, F.R.; Baszler, T.V.; Li, H. Validation of non-nested and real-time PCR for diagnosis of sheep-associated malignant catarrhal fever in clinical samples. J. Vet. Diagn. Investig. 2007, 19, 405–408.

Traul, D.L.; Elias, S.; Taus, N.S.; Herrmann, L.M.; Oaks, J.L.; Li, H. A real-time PCR assay for measuring alcelaphine herpesvirus-1 DNA. J. Virol. Methods 2005, 129, 186–190.
